# A Deep Convolutional Neural Network-XGB for Direction and Severity Aware Fall Detection and Activity Recognition

**DOI:** 10.3390/s22072547

**Published:** 2022-03-26

**Authors:** Abbas Shah Syed, Daniel Sierra-Sosa, Anup Kumar, Adel Elmaghraby

**Affiliations:** 1 Department of Computer Science and Engineering, University of Louisville, Louisville, KY 40208, USA; anup.kumar@louisville.edu (A.K.); adel.elmaghraby@louisville.edu (A.E.); 2 Department of Computer Science and Information Technology, Hood College, Frederick, MD 21701, USA; sierra-sosa@hood.edu

**Keywords:** smart health, Internet of Things (IoT), artificial intelligence, activity recognition, cyber physical systems, fall detection, direction and severity

## Abstract

Activity and Fall detection have been a topic of keen interest in the field of ambient assisted living system research. Such systems make use of different sensing mechanisms to monitor human motion and aim to ascertain the activity being performed for health monitoring and other purposes. Towards this end, in addition to activity recognition, fall detection is an especially important task as falls can lead to injuries and sometimes even death. This work presents a fall detection and activity recognition system that not only considers various activities of daily living but also considers detection of falls while taking into consideration the direction and severity. Inertial Measurement Unit (accelerometer and gyroscope) data from the SisFall dataset is first windowed into non-overlapping segments of duration 3 s. After suitable data augmentation, it is then passed on to a Convolutional Neural Network (CNN) for feature extraction with an eXtreme Gradient Boosting (XGB) last stage for classification into the various output classes. The experiments show that the gradient boosted CNN performs better than other comparable techniques, achieving an unweighted average recall of 88%.

## 1. Introduction

The world is facing an increasing old age population [1] with the group of people aged 65 and older being the fastest growing group. This increase in elderly people is expected to stress healthcare facilities around the world as the elderly are prone to chronic diseases such as heart disease, arthritis, and diabetes [2]. Another aspect of the increased healthcare costs is the increase in the number of falls—in fact, falls are one of the most common injury-causing events in the elderly. It is reported that a third of the people aged 65 and older suffer from falls every year. These falls can result in injuries of moderate to severe nature in the people experiencing the falls and may lead to decreased mobility [3], especially for the elderly [4]. Furthermore, following the initial fall, the likelihood of experiencing additional falls increases [5] and can lead to mental stress in the form of post-fall syndrome [6].

Falls can occur due to a variety of reasons [7]. They can be due to the onset of a sudden health condition such as a heart attack, be a consequence of reduced sensory capability such as impairment of vision, cognition or even due to chronic conditions such as arthritis or Parkinson’s disease. In any scenario, it is pertinent that when a person undergoes a fall, they be provided care as quickly as possible. Providing timely care after a fall may improve post-fall life quality of a patient. Fall detection Systems (FDS) can play a vital role in contributing to the provision of timely care [8,9] by alerting healthcare professionals.

Fall detection falls within the larger umbrella of human activity recognition (HAR). The goal of an FDS is to track a person’s movement and detect when they have fallen in order to warn healthcare staff or other carers. Fall detection systems, for example, are required for elderly persons with cognitive impairments who may not be able to get up after a fall for lengthy periods of time, resulting in pressure sores and other consequences, according to Fleming and Brayne [10].

There are various forms of FDS proposed by researchers. These are Context Aware Systems (CAS) and non-Context Aware Systems (non-CAS). CAS systems include Visual systems, which use a camera or some other visual modality (Kinect based skeletal [11] or depth information [12] ) and ambient systems which make use of sensors placed in an area to sense movement or activities (falls)—these can be position sensors or data from wireless technologies such as WiFi or RF [13]. The drawback of CAS FDS is that they can only be used in a small context, such as a room or a nursing home. This is due to the fact that they are costly to deploy and maintain, as well as the fact that a fixed deployment may limit the user’s freedom of mobility. Furthermore, due to the fundamental nature of the sensing technique, there may be a number of challenges that must be addressed when employing them for fall detection, such as occlusion in Vision-based FDS and spurious sensor triggers in Ambient FDS.

Wearable FDS are a type of non-CAS fall detection system. These systems utilize movement sensors such as accelerometers, gyroscopes and magnetometers, pressure sensors and also various health monitoring sensors [14,15]. The data from these sensors can then be utilized to determine whether or not a fall has happened. Wearable FDS frequently employ many units attached to a user’s body in order to better capture the user’s movement patterns. Wearable FDS, unlike CAS FDS, do not restrict the subjects’ movement and are hence more user pleasant. Furthermore, most wearable FDS sensors, particularly movement sensors, are low-cost and may be found in a wide range of electronic devices, including smartphones and smart watches. Wearable FDS development has therefore been popular for fall detection due to the ease with which such systems can be deployed.

Wearable FDS with accelerometers and/or gyroscopes can be used in conjunction with a person’s phone or as standalone devices affixed to the body. These sensors continuously monitor the movement of a person to gather movement patterns and evaluate the information they collect to ascertain whether or not a fall has occurred. However, before the data recording the movement can be used, first it must be processed, which may include data filtering, windowing and extraction of features. The features are then fed to a decision making system, which indicates whether a fall has taken place or not. In this context, researchers have shown keen interest in machine and deep learning systems as these algorithms can learn nonlinear relationships in the data of activities or falls to identify the event taking place.

This work presents a scheme for performing fall detection considering fall direction and severity as well as activity recognition. Inertial sensor data taken from the SisFall dataset [16] is used to develop the methodology. Data pre-processing is first carried out in terms of windowing and relabeling. Then, data augmentation is carried out for classes which do not have a sufficient number of samples. Lastly, feature extraction is performed along with classification. This work considers fall and activity recognition as a holistic problem, in that different types of falls and activities are considered, thereby producing a more ’complete’ recognition system for use in cyber-physical systems. Moreover, towards this end, a CNN-XGB combination is proposed. Two experiments have been conducted for the designed network, one considering all ADLs individually and the other considering all ADLs as a single class. The results achieved indicate the efficacy of the proposed model for the task for fall detection with direction and severity, as it has been shown to outperform comparable methods for this task.

The paper organization is as follows: a review of the relevant literature is presented in Section 2. The data utilized from the SisFall dataset used in this work is discussed in Section 3 with the methodology being discussed in Section 4. Section 5 elucidates on the experimentation and the results, with a discussion being provided in Section 6 and a conclusion given in Section 7.

## 2. Literature Review

Fall detection comes within the larger umbrella of human activity recognition research. Much of the research in this domain has been performed towards binary fall detection using different machine and deep learning algorithms. However, there have been several approaches presented considering the non-binary case.

Gibson et al. [17] present a fall detection technique that considers fall direction and severity. In their work, Gibson et al. employ multiple classifiers to vote for any of the studied classes. They collect data from accelerometers for Activities of Daily Living (ADLs) and various fall kinds and then utilize a debauchies level-3 wavelet to derive wavelet coefficients from the data. To begin, eight intermediate classes are created from the four output classes, and five different classifiers (ANN, KNN, Radial Basis Function Network (RBF), Probabilistic Principal Component Analysis (PPCA), and Linear Discriminant Analysis (LDA)) are trained to vote for the event to have occurred. Majority voting is used to determine the output class. They use self-collected data for their study. In other work by Hussain et al. [18], fall data from the SisFall dataset is used to develop a fall detection system which can differentiate between the various types of falls present in the dataset. A hierarchical approach is used to carry out this task wherein windowed data from both the accelerometer and gyroscope is passed through a hierarchical classification stage after suitable feature extraction. More recent work in this domain has been performed by Koo et al. [19] and Hsieh et. al [20]. Koo et al. compute statistical features from inertial sensor data and perform tests with a support vector machine (SVM) classifier and an ANN classifier to indicate different phases of a fall. Hsieh et. al use accelerometer data along with a KNN classifier to differentiate between five phases of a fall.

Deep learning methods have gained an increasingly greater use in FDS. Waheed et al. [21] develop a FDS using a Bi-Directional Long Short-Term Memory (Bi-LSTM) network. They consider the binary case of fall and non-fall and perform experiments using the SisFall dataset as well as the UP-Fall dataset [22]. Their network consists of eight layers in total. Two Bi-LSTM layers and two Fully Connected Layers with dropouts are used for regularization. Training is performed with creating missing values in the data to introduce noise tolerance. A modified AlexNet [23] has been used in [24] by Alarifi et al. They collect tri-axial data from inertial measurement sensors consisting of accelerometer, gyroscope, and magnetometer at six different positions on a subjects body. A total of 16 activities of daily living and 20 falls were recorded by them. Feature extraction is then performed in terms of various statistical measurements as well as frequency analysis. This is followed by principal component analysis and then passed on to the classification stage consisting of an optimized AlexNet ConvNet.

One proposed method to perform combined activity recognition and fall detection has been presented in [25] by Li et al. In this, Li et al. use multi-modal sensor fusion and a Bi-LSTM classification network to differentiate between five activities of daily living and a fall. The sensors they use are an inertial sensor placed on the wrist, waist, and ankle as well as a radar sensor. After pre-processing both the inertial measurement and radar signals, various statistical and moment computations were performed to be used as features. These were passed on to the multilayer Bi-LSTM network after feature selection to determine the output class. Another method addressing the combined activity and fall detection problem has been proposed by Chelli et al. [26]. They perform experiments using two publicly available datasets and work on accelerometer and angular velocity data. Statistical, time, and frequency domain features are computed from the sensor measurements. Experiments were conducted with four machine learning algorithms: ANN, KNN, quadratic SVM (QSVM) and ensemble bagged tree (EBT). From their experiments they find that QSVM and EBT produce the best results. One thing to note is that both [25] and [26] consider fall as a single category rather than considering falls as a detailed problem (direction and/or severity) in itself.

More recent work by We et al. [27] also considers activity recognition and fall detection together. They use inertial measurement sensor data from two datasets, the MobiAct dataset [28] and the SmartFall dataset [29]. The MobiAct dataset contains data from four falls and nine activities of daily living, whereas the SmartFall dataset has non-fall and fall recordings. In their experimentation, they compare the performance of different machine learning models and several deep learning models, including a CNN, LSTM, and CNN-LSTM combination and Gated Recurrent Units (GRU). The machine learning models are trained by computing time and frequency domain features, whereas the deep learning models are trained using raw sensor data. They find that the GRU designed by them, consisting of two GRU units followed by a softmax classification layer, is the best performing model. Another deep learning approach utilizing sequential modeling for a fall detection system that also considers ADLs has been presented by Sengül et al. [30]. They collect their own data for two types of falls and four activities of daily living. After data augmentation on the minority classes, they use a Bi-LSTM for classification.

As can be noted from the literature discussed, while fall detection has been studied in more depth as a binary detection system (fall and non-fall), relatively less research has been done on the more complex case of non binary fall detection. To contribute towards this end, in Syed et al. [31], the problem of fall detection while considering direction and severity was addressed. Here, several time and frequency descriptors were computed from IMU sensor signals taken from the SisFall dataset and an SVM classifier was used for indicating the impacts of falls as well as their direction. Further work in Syed et al. [32] followed the work of [25,26] by considering falls and ADLs together. Herein, falls constituting different directions and severity levels were considered along with four common ADLs and a hierarchical classification scheme was developed. Following this work, this paper presents a convolutional neural network architecture with an XGB stage for performing ADL and fall detection as a combined problem, thereby proposing a holistic solution for ambient assisted living deployments. The network is trained using data from the publicly available SisFall dataset and an improvement in the unweighted average recall (UAR) is achieved compared to previous approaches.

## 3. Data

The Universidad de Antiquia provided the SisFall dataset to aid research in the development of fall detection systems. The provided dataset consists of recordings of 38 volunteers performing various types of falls and ADLs. In total, there are 19 ADLs and 15 falls and for each ADL and fall, bar walking and jogging, 5 trails were performed by each volunteer. This brings the total number of recordings for ADLs to be 2707 and for falls to be 1798. The data was collected from the sensors with a 200 Hz sampling rate while the belt containing the sensor unit was attached to the persons waist. The sensor used was an IMU with two accelerometers and a single gyroscope.

The SisFall data is utilized in this study as it has been the dataset of choice for several previous research approaches addressing the subject of fall detection [33,34,35] given that it incorporates volunteers from a large range of ages (19–75 years), consists of both male and female participants (19 each to make 38 in total), and also because the type of activities and falls recorded are decided based on real world situations. However, since the SisFall dataset has been produced for fall detection in general and not specifically for fall direction and severity analysis, the original labeling of the dataset had to be modified. This has been detailed in Table 1.

Looking at the table, it can be observed that the various activities in the SisFall dataset have been grouped into four major activities of daily living, which are the ADLs of Walking (W), Jogging (J), Sitting/Sit (S) and Standing (SB). These larger groups of ADLs contain samples from more granular activities, for, e.g., the group Walking, which consists of samples of slow walking and fast walking as well as walking over stairs. There are some activities that have not been considered in this work. Examples of such activities include entering in to and exiting out of a car. Table 1 also shows the labeling used for falls. The falls have been labeled simultaneously on their direction as well as severity. While the original labels from the dataset contained information for most falls for direction, the approach followed by Gibson et al. [17] was used to determine the severity of falls. According to the practice followed by them, all falls where in some support was used to soften the impact of the fall were considered as soft falls whereas all falls where the subject fell directly were classified as hard falls. This resulted in six classes for fall types with hard and soft for imapact and forward, backward and lateral for direction. These are Forward Soft Falls (FSF), Forward Hard Falls (FHF), Backward Soft Falls (BSF), Backward Hard Falls (BHF), Lateral Soft Falls (LSF), and Lateral Hard Falls (LHF). In order to better illustrate the labeling process of the falls, plots of the sample recordings have been shown in Appendix A.

## 4. Methodology

**Figure 1:**
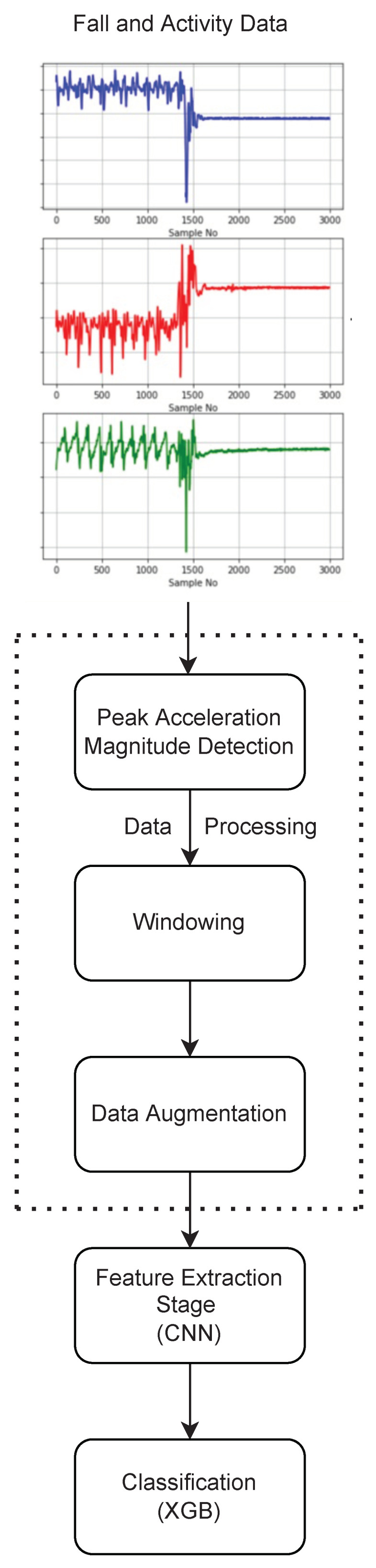
Hierarchical classification scheme for ADL and Fall detection.

The proposed scheme follows a typical deep learning solution framework. First, inertial sensor data from the IMU sensors contained within the SisFall dataset is pre-processed to extract windowed segments, then data augmentation is performed for minority classes, followed by feature extraction and then classification. This has been illustrated in Figure 1 and a discussion is provided for each of the steps in the proceeding sections.

### 4.1. Data Pre-Processing

Before the IMU sensor recordings can be used for ADL and Fall detection, raw sensor measurements need to be suitably processed. In this work, data pre-processing consists of two steps, the first is the extraction of uniform sized windows from the IMU recordings and the next is data augmentation.

#### 4.1.1. Windowing

Due to the nature of the recording scenario, the duration of the records in the SisFall dataset varies from 12 to 100 s. However, the subsequent feature extraction and classification stage requires the provided input be of uniform duration. In order to this, windowing was performed on the IMU data. To do this, first the root sum of the squared value of the acceleration components, called the support magnitude vector (SMV), was computed for each data point in the signal recordings. The SMV can be calculated mathematically as,

(1) $$SMV_{j}=\sqrt{{\left|A_{x_{j}}\right|}^{2}+{\left|A_{y_{j}}\right|}^{2}+{\left|A_{z_{j}}\right|}^{2}}$$

In the mentioned equation, $SMV_{j}$ indicates to the SMV computed for the acceleration data-point j in an activity or fall recording. After the SMV values for all data points has been computed, the data-point location which gives the peak value for the current sample/recording is then found. This location is used as the mid-point to extract a window of duration n (3 in this case) seconds from the sample/recording. This windowing approach has been followed for all considered ADLs and Falls in the SisFall dataset except for the D01, D02, D03 and D04 activities. The reason for this is that the duration of these activities was 100 s and they had fewer trials. These contain the activities of continuous walking or jogging. In order to include a sufficient number of samples from these activities, a uniform sliding window scheme with no overlap was used to form individual training samples of these activities from the complete recordings. These approaches for windowing have been used by other authors in [36]. In this manner, windows were extracted for the gyroscope sensor measurements for each label as well. Furthermore, it should be noted that data from only one accelerometer was used so that the total vector size for every data-point in the label sample was 6 values, 3 for the tri-axial accelerometer, and 3 for the tri-axial gyroscope. The data from both IMU modalities (accelerometer and gyroscope) is used for the problem at hand since previous studies [21,32] have shown that a combination of these two modalities provides better performance compared to any of them individually.

#### 4.1.2. Data Augmentation

**Figure 2:**
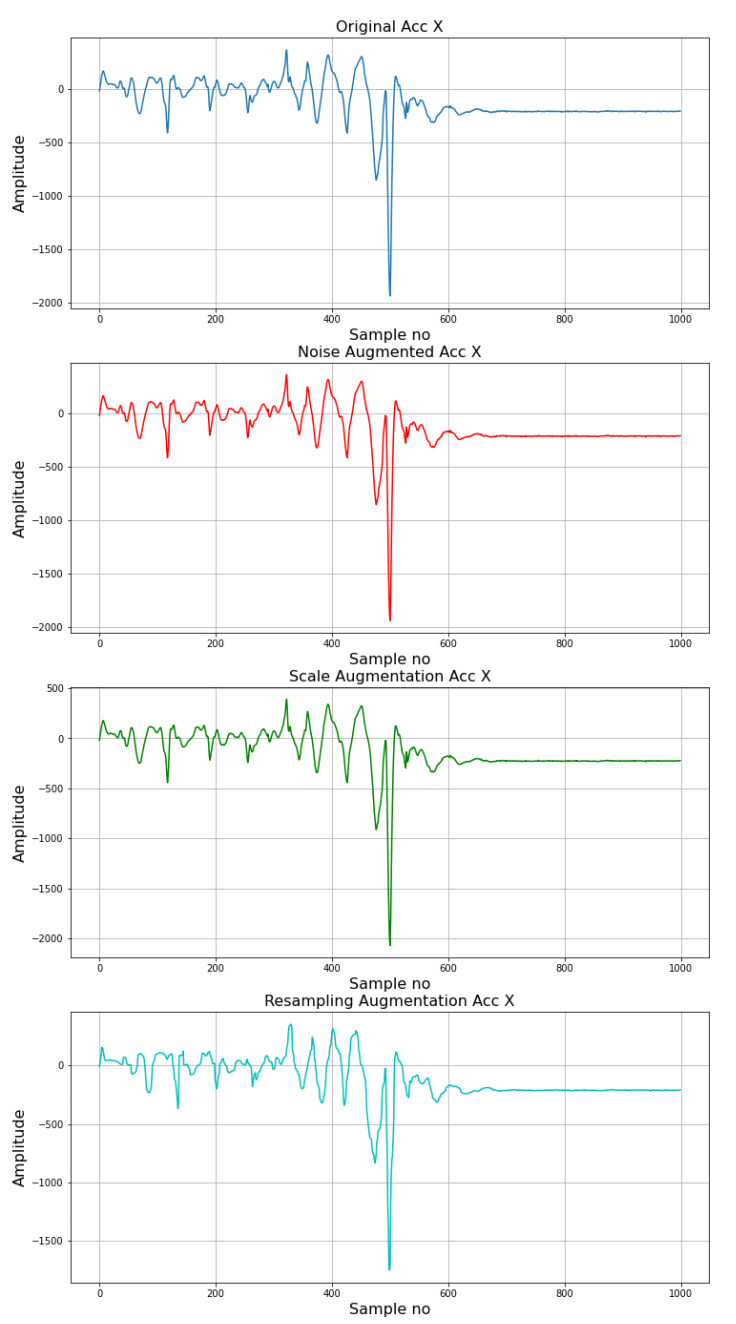
Illustration of data augmentation. (X component of the accelerometer, lateral fall).

The use of deep learning methods require a suitable amount of data to be present for them to learn the data pattern sufficiently well. Unfortunately, due to the nature of the problem considered, the relabeled data from the SisFall dataset contains a reduced amount of data for some of the classes, especially fall classes and also for the ADL of Standing as observed from Table 1. In order to alleviate this shortcoming, data augmentation was employed to increase the number of samples from these classes. Three augmentations were performed for each of the extracted recordings for the classes SB, FHF, FSF, BHF, BSF, LHF, and LSF. These were the addition of noise, scaling, and resampling after interpolation [37]. For augmentation with noise, white Gaussian noise was added to the extracted windows of the considered classes. The noise was generated using a standard deviation equal to 0.01. The addition of the noise simulates measurement noise during recording, which might be encountered when IMU-based fall detection systems are employed. For scale-based augmentation, the original extracted window was multiplied by a random number from the uniform distribution between 0.8 and 1.2. By doing so, changes in amplitude over the same type of activity/fall are incorporated. This could indicate a change in fixation (loosening, etc.) of the sensing unit to a subject or their different physique and subsequent fall intensity response. Lastly, in order to incorporate sampling inconsistencies, the windows are first upsampled and then downsampled. This was done using a scale of 10. With this strategy, each original window produced three additional windows. An illustration for the results of the augmentation process for an X-axis accelerometer measurement for a lateral fall has been shown in Figure 2.

### 4.2. Feature Extraction and Classification

**Figure 3:**
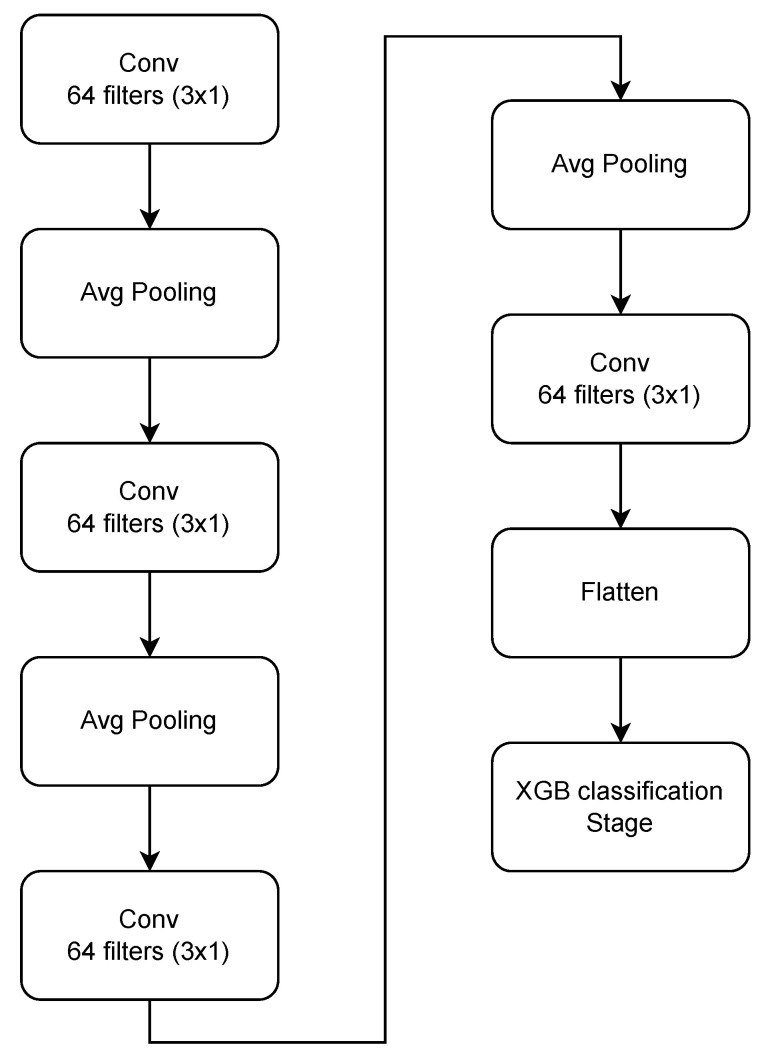
CNN network for feature extraction and XGB classification stage.

The aim of feature extraction for a classification problem is to produce a representation of the input that can be better used to indicate to the output class. In this regard, research in the area of fall detection with inertial sensors has made use of different types of hand crafted features such as statistical, time and frequency domain, as well as wavelet transforms [38,39,40,41]. Convolutional Neural Networks (CNNs or Covnets) are a set of neural networks developed following the visual cortex within the brain [42]. CNNs perform operations on inputs by introducing convolutions of several filters with learnable weights to gauge the importance of each datapoint in the input. The layers containing the filters and to which the input is provided are called the convolutional layers. Through these learnable weights, CNNs are able to capture temporal and spatial dependencies of the input. Moreover, using the same filters for different inputs reduces the number of parameters as the weights are reused. This allows CNNs to develop a deeper understanding of the provided input compared to typical multilayer perceptron models. CNNs have revolutionized the field of computer vision where they have been used for a variety of tasks such as classification, object detection, segmentation, and object counting [43,44] and they have also successfully been used for applications within the speech and other time series signal application domain [45,46,47]. In this paper, rather then using hand crafted features, a CNN has been used to perform feature extraction in order to take advantage of the spatial and temporal dependency capturing capabilities of CNNs. CNNs are usually comprised of several convolutional (Conv) layers. Within a multilayer CNN, the earlier Conv layers capture low level features from the input with more complex features being computed by the successive layers. In addition, CNNs may employ pooling layers between convolutional layers so as to reduce the size of the input passed on to successive layers and therefore reduce computation. The proposed network along with the XGB classification stage is illustrated in Figure 3.

As seen from the figure, the network consists of four Conv layers of 64 filters each. These layers have filters of size 3 × 1 and are used to extract features from raw inertial sensor measurements. Each Conv layer is followed by a Relu activation function, which applies a non-linearity to the output of the Conv layers. To condense the output of the first three Conv layers, a pooling layer is utilized. Instead of max pooling, average pooling is used in this network. Max pooling picks out the largest value of the patch of data being observed currently. In contrast, average pooling uses the average of the data being observed. Average pooling has been successfully used in place of max pooling in a variety of scenarios [48,49]. Normalization is performed using a batch normalization layer for each Conv layer. The output of the last Conv layer are feature maps derived from the input raw inertial sensor measurements from both the accelerometer and the gyroscope. Classification is carried out by using an eXtreme Gradient Boosting (XGB) classifier. The output from the CNN is first flattened and then provided as an input to the XGB stage. The parameters of the XGB classifier are tuned through a parameter search over a range of values. We make use of an XGB classifier due to its suitability for a large dimensional input which results from the flattened CNN output.

## 5. Experimentation and Results

In order to use the proposed CNN for feature extraction, it must first be trained accordingly. In order to train the network, a fully connected layer with a softmax output was added as the final stage to serve as the intermediate temporary output determinant stage. The windowed data from the SisFall dataset was divided into three sets: train, validation, and test in a stratified manner. A learning rate of 0.01 was used for the network with a batch size of 20 and the stochastic gradient descent was used as the optimizer. Moreover, the metric chosen was the average of the recall scores of all classes together, also called the unweighted average recall (*UAR*). The recall is considered, as one wants the system to correctly classify as many positive samples for every class as possible. The final network was determined using early stopping. The unweighted average recall (*UAR*) can be computed as,

(2) $$UAR=\sum_{n=0}^{k}\frac{\left(\frac{TP_{n}}{TP_{n}+FN_{n}}\right)}{k}$$

where *k* stands for the number of classes and *TP_n_* stands for the number of True Positive samples in the nth class and *FN_n_* stands for the number of False Negative samples in the nth class. Therefore, the average of the individual recall scores from all classes was aimed to be maximized.

Data from the training set was provided to the CNN network after performing data augmentation on the minority classes, during training, the validation set was used to observe the performance of the network and determine the best performing instance. Once training was finished, the last fully connected classification layer was removed and replaced by an XGB classification stage. To train the XGB stage, the weights of the final best performing CNN model were loaded in to CNN layers of the network and input samples were then passed through them as before. Using the output of the CNN stage as an input for the XGB, a search was then carried out to determine the optimal parameter values. After training of the XGB stage, the completely trained CNN-XGB model was tested using the test set. The results of this experiment have been shown in Table 2.

## 6. Discussion

From Table 2, it can be observed that an unweighted average recall (UAR) of 88.25% was achieved on the test set by the proposed scheme. This is an improvement of nearly 4% compared to the previous work by the same authors [32]. When looking at the recall achieved for the individual activities, it can be observed that the best recognized activities are W and J, with each achieving a recall of around 97%. The worst performance in terms of ADLs was achieved for the activity SB for which a recall of 83.78% was attained. Considering the case of falls, out of the six falls, the worst recall score of 75% was achieved for BHF and LHF, whereas the best recall score was of 95.83% was achieved for BSF and LSF. The fall class FSF was also not identified well, achieving a recall score of 77.08%. Furthermore, as observed from the table, a high value of specificity was obtained for all ADLs and Falls, indicating correct determination of negative samples for each class as well. Investigating the performance of the network from the confusion matrix, it was observed that the worst performing activity SB was equally as confused as S and W. This can be attributed to the fact that the SB activity includes slight bending, which could lead to confusion for the classifier. For the worst performing falls, it was observed that BHF was confused with LHF and LSF whereas LHF was confused with FHF and FSF. The confusion between the falls is apparent from plots shown in Appendix A where BHF has very similar accelerometer signal values to these classes.

**Figure 4:**
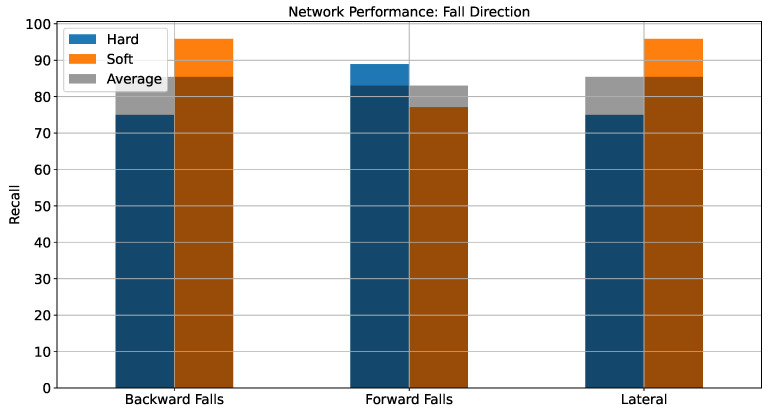
Network performance for different fall directions.

In order to better understand the performance of the network for the various fall detection types from Table 2, Figure 4 illustrates the performance of the network in fall detection for the three directions considered—regardless of severity. It can be observed that falls in backward and lateral directions are determined with equal effectiveness, an average recall score (UAR) of 85.42% was achieved for both cases, whereas for the forward direction, the UAR was 82.99%. As can be seen, there is a only a small difference (3.43%) between the achieved UARs.

**Figure 5:**
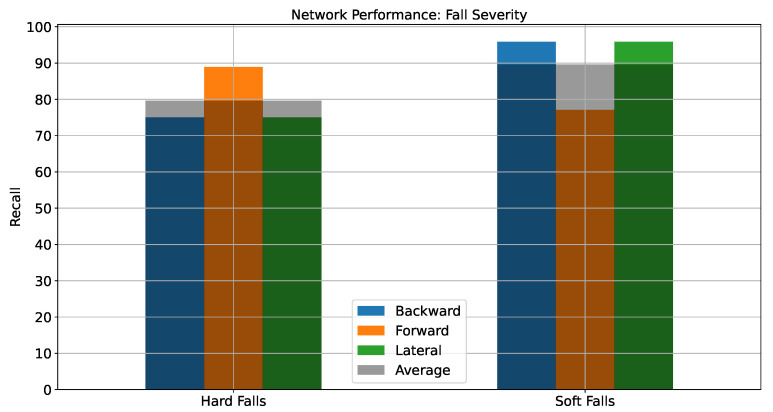
Network performance for different fall Severity.

The performance of the network for fall severity has been illustrated in Figure 5. It can be observed that the UAR for hard falls is 79.63% and that for soft falls is 89.58% indicating a better performance by the network at detecting soft falls compared to hard ones.

As another test of the network performance, Table 3 presents network performance when the ADLs are considered a single class. It can be observed that grouping the ADLs increases the overall performance of the network with an average recall of 88.89%. The ADLs are detected with a high value of the recall 100%. Moreover, in the recall for all the fall types bar, the class LHF is above 80%. There has been a reduction in performance for the falls in the lateral directions. However, when comparing the average recalls scores for the falls only, in Table 3, an average recall of 87.04% is achieved, whereas in Table 2 it was 84.60%, which is lower than the current experiment. A caveat here is the below par performance for the LHF class at 66.67%, which is the lowest recall score achieved for any class in both experiments for the designed network.

In order to further test the performance of the developed CNN-XGB scheme, tests were performed with the CNN network architecture presented in [36] and the work in [27], where sequential modeling is performed. These methods were adapted for the task at hand. We choose the technique of [36] as the authors provide very good results for fall detection using the SisFall dataset using a deep learning model. The work of [27] is chosen, as they consider the combined problem of fall detection and activity recognition and are therefore similar in terms of application. The results for the performance of both the considered techniques in comparison to the method presented in this paper is shown Table 4. The recall scores have been presented for the techniques. The mean recall scores achieved are 85.69% for [36] and 80.07% for [36] compared to more than 88% for the proposed scheme. It can be observed from Table 4 that the proposed CNN-XGB combination outperforms the work of Casilari et al. [36] in six out of the seven output classes for recall while achieving a similar performance for the classes of BHF, LHF, and S. In comparison to the work of [27], it can be observed that the proposed method performs better for 8 of the 10 classes whereas performing lower for the classes FSF and J, the difference being the highest for FSF. The table also highlights the capability of CNNs for feature extraction purposes as the two approaches based on CNNs perform much better than the non-CNN approach for classes of BHF and LHF, for which the other method provides subpar results.

Regarding the computational complexity at inference time of the four algorithms in Table 1, for the two CNNs, the computational complexity of one CNN layer is O(kndf) [50] where k is the kernel size, n is the input length, d is the depth of the layer, and f the filter size. Since the model is sequential, the contribution of other layers is additive. Moreover, for our work, the added XGB stage adds a complexity time of O(td), where t is the number of trees in the model, and d is the output vector size of the CNN stage. The work of [27] uses GRUs along with a fully connected last stage, therefore the computational complexity at inference is O(ndu) [50] where n is the input sequence length, d is the depth (number of features) and u the number of units for the layer. From this, it can be concluded that the CNN models are more intensive in terms of the number of operations being performed. However, it must be noted that sequential learning models typically have higher memory requirements and have been found to be slower than CNNs for a variety of different tasks [51].

## 7. Conclusions

Activity and fall detection has been a topic of keen interest in the field of ambient assisted living system research. Such systems make use of different sensing mechanisms to monitor a subject’s motion and aim to ascertain the activity being performed for health monitoring and other purposes. Towards this end, fall detection is an especially important task as falls can lead to injuries and sometimes even death. Even though there are various modalities used for the development of activity of daily living and fall detection systems, systems making of movement sensors present within inertial measurement units are popular due to the low cost of deployment and ease of use. In this work, a fall detection and activity recognition system that not only considers various activities of daily living but also considers detection of falls while taking into consideration their direction and severity has been proposed. Inertial Measurement Unit (accelerometer and gyroscope) data from the SisFall dataset is first windowed into non-overlapping segments with a duration of 3 s. After suitable data augmentation for the minority classes, the windowed segments are passed to a Convolutional Neural Network for feature extraction. The CNN is trained to maximize the unweighted average recall for the validation partition. Once the CNN is trained, an XGB last stage is used for classification. Experiments conducted on the test set achieve an unweighted average recall of 88%. In comparison with other techniques used for this task, the proposed scheme produces sufficiently better results, thereby demonstrating the efficacy of the proposed method.

Following from the work presented, there are various directions for further work. From a sensor modality perspective, the addition of additional sensor modalities might help improve performance for the worst detected classes. Various authors have incorporated medical or pressure sensors in their fall detection systems. Data from these sensors can be used together as an input to a deep learning network. This additional information gathered for subjects while undergoing a fall could also describe valuable health parameters that can be used for diagnosis or early detection of ailments, which might be the underlying cause of falls.

Another aspect of future work would be in the domain of deep learning algorithm development. In this regard, transformer networks have been found to be very useful for natural language processing applications and have been getting attention in applications relating to time series signals as well [52]. Attention networks have the capability to assign weights important parts of a signal more and therefore allow for potentially better performance than traditional deep learning approaches. Another work opportunity in the data science domain would be the assessment of this problem considering a data-centric approach, where, in contrast to a model-centric approach where the focus is on developing the best model, a data-centric approach focuses on working with data to improve application performance using systematic procedures for labeling, augmentation, etc. Such systematic procedures and qualitative data analysis would allow for cross-dataset algorithm deployment as well.

## Figures and Tables

**Table 1:** ADL and Fall Labels used for SisFall Recordings.

SisFall			Assigned	Assigned
Activity/Fall Code	Brief Description	Trials	Activity/Fall Name	Activity/Fall Label
D01	Walking (slowly)	1	Walking	W
D02	Walking (quickly)	1	Walking	W
D03	Jogging (slowly)	1	Jogging	J
D04	Jogging (quickly)	1	Jogging	J
D05	Walking stairs (slowly)	5	Walking	W
D06	Walking stairs (quickly)	5	Walking	W
D07	Sit on chair (slowly)	5	Sit	S
D08	Sit on chair (quickly)	5	Sit	S
D09	Sit on low height chair (slowly)	5	Sit	S
D10	Sit on low height chair (quickly)	5	Sit	S
D11	Sitting (collapse down)	5	Sit	S
D12	Sitting (lying slowly)	5	Sit	S
D13	Sitting (lying quickly)	5	Sit	S
D15	Standing	5	Standing	SB
D16	Standing	5	Standing	SB
F01	Fall Forward (slip)	5	Forward Hard Fall	FHF
F02	Fall backward (slip)	5	Backward Hard Fall	BHF
F03	Lateral fall while walking (slip)	5	Lateral Hard Fall	LHF
F04	Fall forward while walking (trip)	5	Forward Hard Fall	FHF
F05	Fall forward while jogging (trip)	5	Forward Hard Fall	FHF
F06	Vertical fall while walking (faint)	5	Forward Soft Fall	FSF
F07	Fall while walking (faint)(dampened with support)	5	Lateral Soft Fall	LSF
F08	Fall forward while trying to get up	5	Forward Soft Fall	FSF
F09	Lateral fall while trying to get up	5	Lateral Soft Fall	LSF
F10	Fall forward when trying to sit down	5	Forward Soft Fall	FSF
F11	Fall backward when trying to sit down	5	Backward Soft Fall	BSF
F12	Lateral Fall when trying to sit down	5	Lateral Soft Fall	LSF
F13	Fall forward while sitting (fainting/sleeping)	5	Forward Soft Fall	FSF
F14	Fall backward while sitting (fainting/sleeping)	5	Backward Soft Fall	BSF
F15	Lateral while sitting (fainting/sleeping)	5	Lateral Soft Fall	LSF

**Table 2:** ADL and Fall detection results.

Activity	Precision (%)	Sensitivity/Recall (%)	Specificity (%)	F1-Score (%)
BHF	100	75.00	100	85.71
FHF	76.19	88.89	99.01	82.05
LHF	75.00	75.00	99.71	75.00
BSF	95.83	95.83	99.90	95.83
FSF	90.24	77.08	99.60	83.15
LSF	86.79	95.83	99.30	91.09
J	96.71	96.71	99.01	96.71
S	96.77	96.77	99.57	96.77
SB	91.18	83.78	99.70	87.32
W	97.21	97.63	97.77	97.42
Average	90.59	88.25	99.36	89.11

**Table 3:** Individual falls vs. ADL.

Activity	Precision (%)	Sensitivity/Recall (%)	Specificity (%)	F1-Score (%)
BHF	100	91.67	100	95.65
FHF	85.37	97.22	99.41	90.91
LHF	72.73	66.67	99.70	69.57
BSF	100	95.83	100	97.87
FSF	92.86	81.25	99.71	86.67
LSF	89.58	89.58	99.50	89.58
ADL	99.54	100	97.78	99.77
Average	91.44	88.89	99.44	90.02

**Table 4:** Comparison to other works.

Activity	Sensitivity/Recall (%)		
	Work of [27]	Work of [36]	Proposed Work
BHF	58.33	75.00	75.00
FHF	83.33	80.56	88.89
LHF	41.67	75.00	75.00
BSF	87.50	87.50	95.83
FSF	81.25	72.92	77.08
LSF	81.25	93.75	95.83
J	98.77	96.30	96.71
S	88.71	96.77	96.77
SB	83.78	81.08	83.78
W	96.12	95.04	97.63
Average	80.07	85.39	88.25

**Figure A1:**
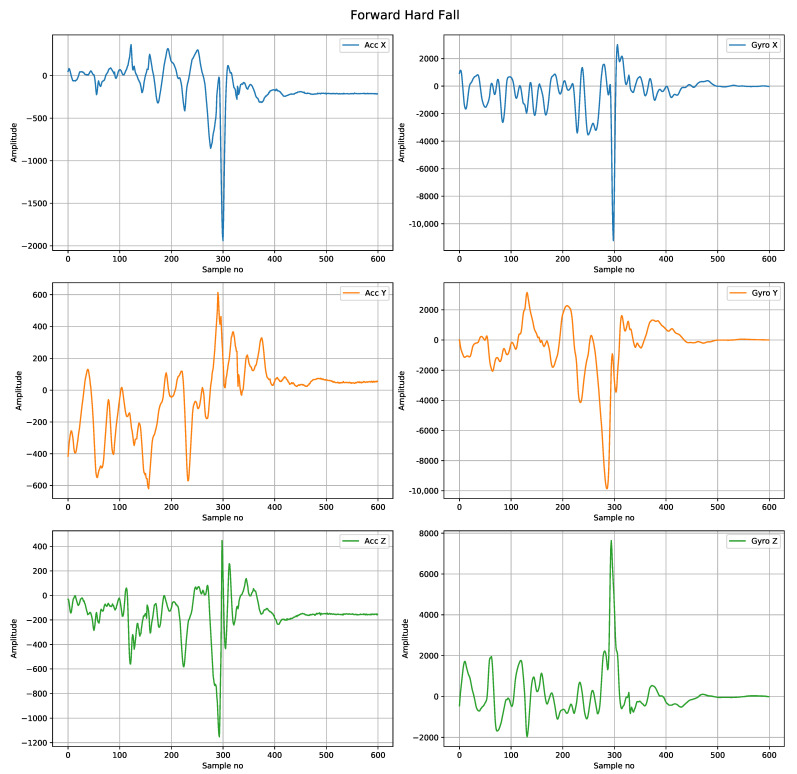
Accelerometer and gyroscope measurements: forward hard fall.

**Figure A2:**
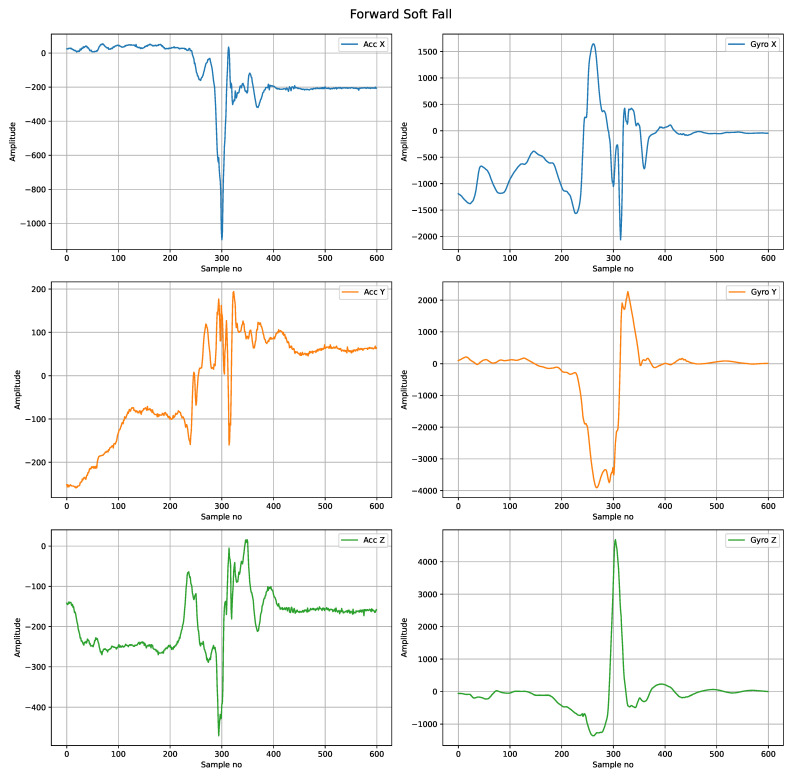
Sample of forward soft fall.

**Figure A3:**
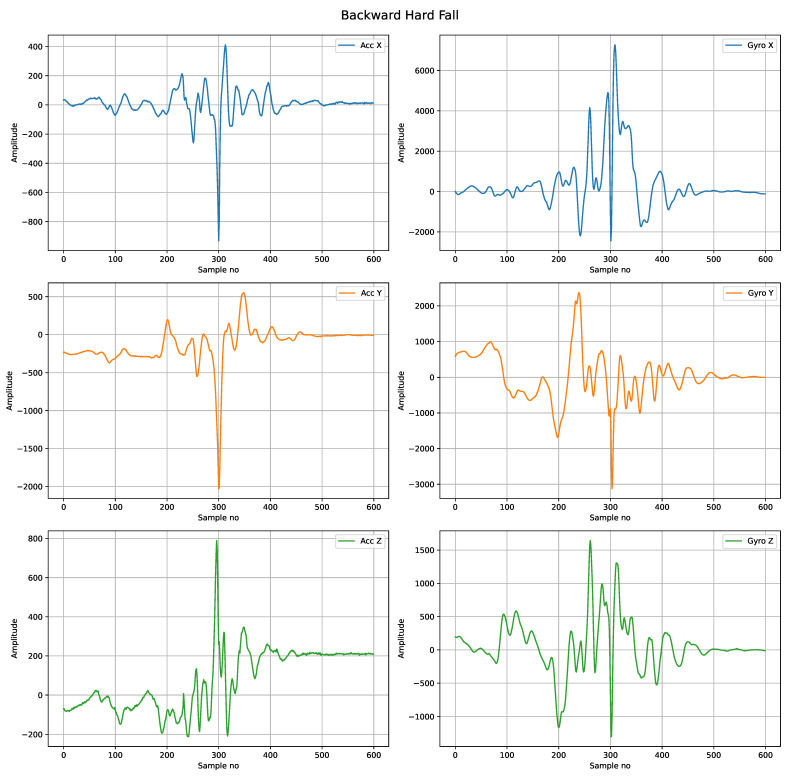
Accelerometer and gyroscope measurements: backward hard fall.

**Figure A4:**
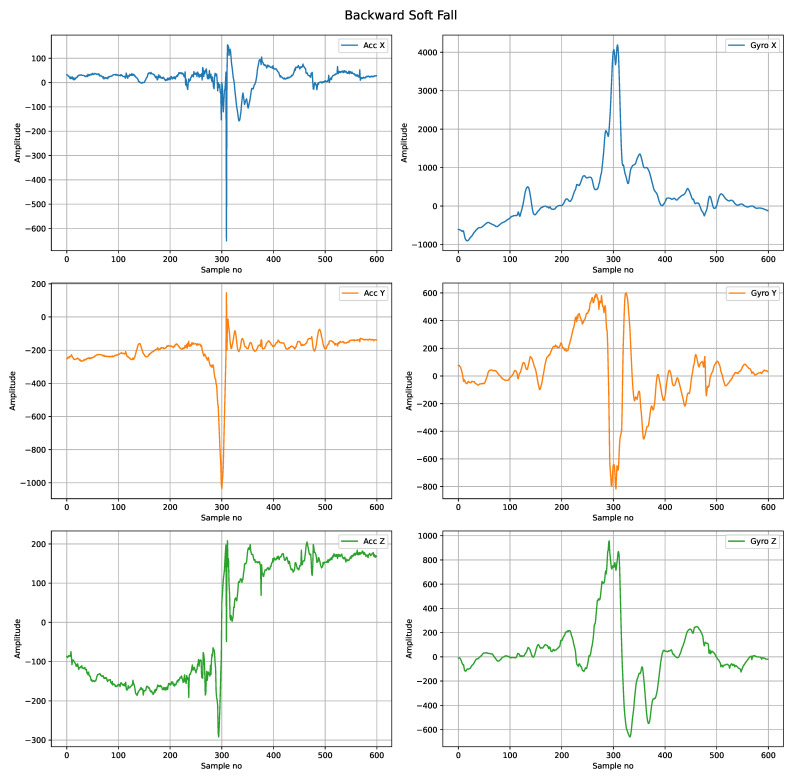
Accelerometer and gyroscope measurements: backward soft fall.

**Figure A5:**
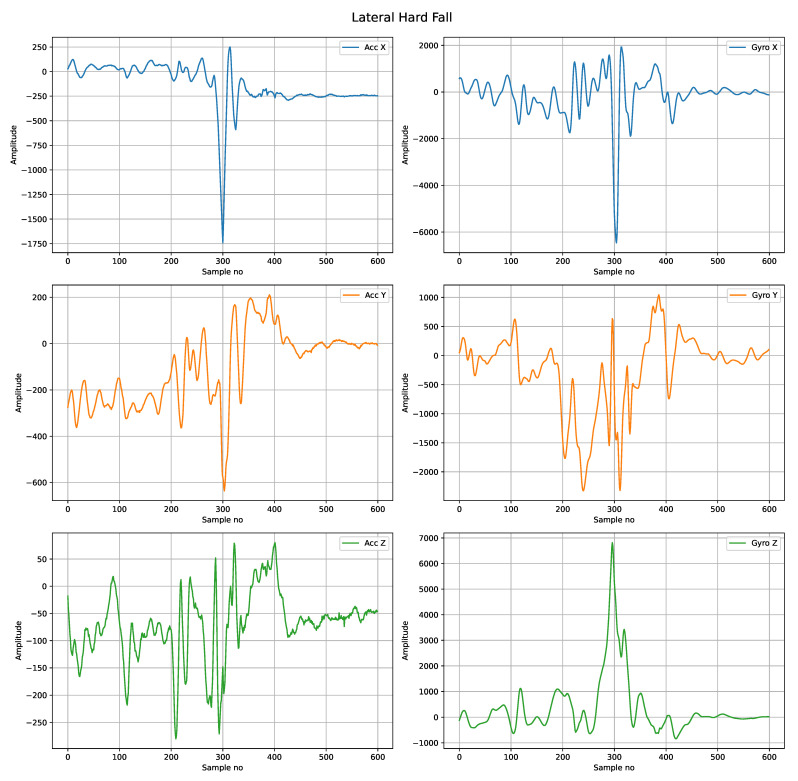
Accelerometer and gyroscope measurements: lateral hard fall.

**Figure A6:**
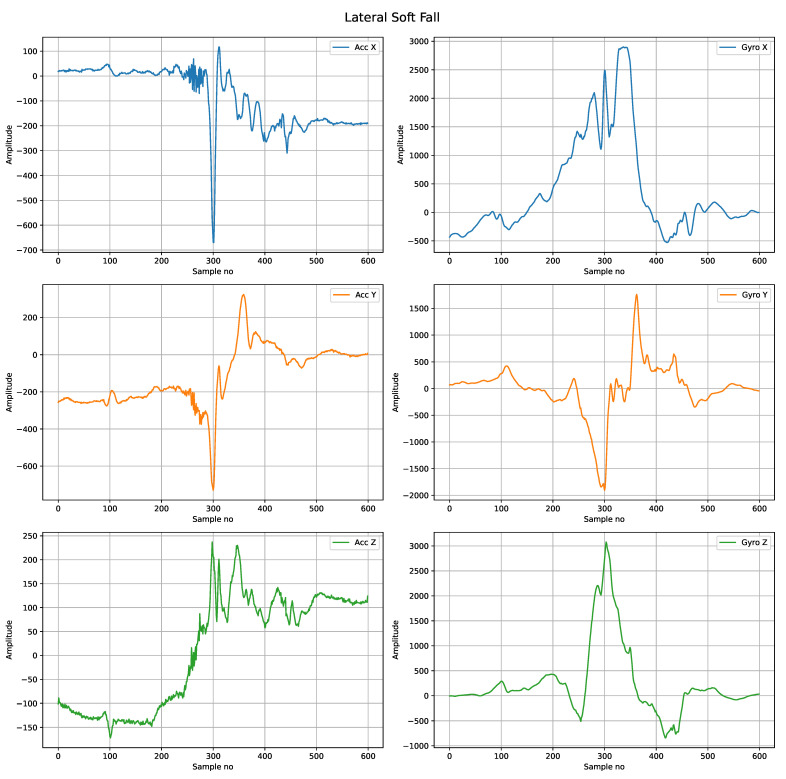
Accelerometer and gyroscope measurements: lateral soft fall.

## Data Availability

The dataset used in this work is publicly available at: http://sistemic.udea.edu.co/en/research/projects/english-falls/ accessed on 15 January 2022.

## Appendix A

Sample plots of the windowed accelerometer and gyroscope measurements of different fall types.
